# Mass balance of complementary metasomatic processes using isocon analysis

**DOI:** 10.1016/j.mex.2021.101609

**Published:** 2021-12-21

**Authors:** Evgeniy N. Kozlov, Ekaterina N. Fomina

**Affiliations:** Geological Institute, Kola Science Centre of the Russian Academy of Sciences, Apatity 184209, Russia

**Keywords:** Isocon analysis, Mass balance, Metasomatism, Carbonatites

## Abstract

This study develops a method to estimate the redistribution of elements during several associated metasomatic processes simultaneously involved in the formation of a geological complex*.* The method is based on classical mass balancing using isocon analysis. The proposed approach is applicable if geological prerequisites indicate that (a) the metasomatic rocks inherited some components (*x, y*, etc.) from one of the earlier rocks of the studied complex; (b) these components have been transported by a fluid; and (c) this saturated fluid has produced several varieties of metasomatic rocks rich in components *x, y*, etc. In the case of the specified background, the proposed method estimates the mass proportions between the source rock and the varieties of the resulting metasomatic rocks. We present the geological profile of the processes to which the proposed method is applicable, the mathematical model of the method, examples of the application and interpretation of the results, and geological criteria to verify the obtained model results. In brief,

• This study introduces a method for calculating the mass proportions between the source rock and metasomatic rocks formed from the material remobilized from the source rock;
• The paper considers the applicability limits of the method, exemplifies the interpretation of the results, and shows the methods for monitoring the correctness of these results.

## Graphical abstract

**Figure:**
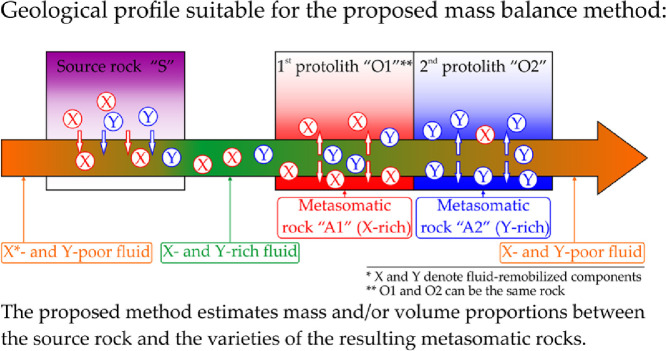

**Specifications table**

**Table:** 

**Subject Area**	Earth and Planetary Sciences
**More specific subject area**	Geochemistry and Petrology
**Method name**	Mass-balance of complementary metasomatic processes
**Name and reference of original method**	“Gresens method” or “isocon analysis”: • R.L. Gresens, Composition-volume relationships of metasomatism, Chem Geol. 2 (1967) 47–65. https://doi.org/10.1016/0009-2541(67)90004-6. • J.A. Grant, The isocon diagram; a simple solution to Gresens’ equation for metasomatic alteration, Econ Geol. 81 (1986) 1976–1982. https://doi.org/10.2113/gsecongeo.81.8.1976.
**Resource availability**	N/A

## Method details

### Background: isocon analysis

Metasomatism is the process of altering the composition of a rock by adding or subtracting chemical elements [1,2]. This is a systematic process that occurred pervasively in various geological settings; therefore, it has played a fundamental role in crust formation. During metasomatism, chemical elements are intensely separated and redistributed between the so-called “metasomatic zones” [3]. Sometimes it results in forming ore concentrations. In this regard, metasomatic processes attract the keen interest of many researchers. One of the practical tools for studying metasomatism is the method proposed by R.L. Gresens [4] and later simplified by J.A. Grant [5]. The latter modification is called isocon analysis. It has proven to be effective for various geological objects [6], and the number of references to the article [5] at the time of this writing exceeds 800. The summary of the method is as follows. For the protolith rock subjected to metasomatic impact, the change in the mass of an *i* (Δ*M_i_*) component is described as [5]:

(1) $$\Delta M_{i}=\left(\frac{M^{A}}{M^{O}}\;\times \;C_{i}^{A}-\;C_{i}^{O}\right)\;\times \;M^{O},$$

where *М* is the mass and *C_i_* is the concentration of components in the protolith (O) and altered rock (A). The basic formula determining the concentration of *i* component in the altered rock is [6]:

(2) $$C_{i}^{A}=\frac{M^{O}}{M^{A}}\times \left(\;C_{i}^{O}+\Delta C_{i}\;\right).$$

For immobile species, this equation is transformed as follows:

(3) $$C_{i}^{A}=\frac{M^{O}}{M^{A}}\times C_{i}^{O}.$$

In the $\;C_{i}^{O}$ vs. $C_{i}^{A}$ plot, the points of immobile components are located on the “zero mobility” line, which has the slope of *M*^O^/*M*^A^ and starts at the origin of ($C_{i}^{O}$ = 0, $C_{i}^{A}$ = 0). This line is called the isocon [7]. Its slope indicates the total change in mass relative to the *M*^O^. According to [6], the $C_{i}^{A}/\;C_{i}^{O}$ data clustering or a best fit of data forming a linear array through the diagram origin apply to determine the isocon slope. Another way to achieve this goal is to introduce one of a priori assumptions about (1) the immobility of certain components, (2) the mass constancy, or (3) the volume constancy during alterations [6]. Manual calculations for performing isocon analysis are too laborious, but some computer programs can automate this method (e.g., [8], [9], [10]). In our calculations, the user-friendly GeoIso software [11] was utilized. Most often, the isocon analysis is used to explain how one rock was transformed into another. This study focuses on a more complicated problem of element redistribution in the course of several conjugate processes simultaneously involved in the formation of a geological complex.

### Mathematical description of the mass balance of complementary metasomatic processes

Here, we present a schematic exemplification of a geological object for which the mathematical model has been developed. Based on geological prerequisites, a certain source rock (S) was exposed to metasomatic alterations. During metasomatic processing, a fluid removed *x* and *y* components from this source rock and transferred them to other (O1, O2, etc.) rocks. Further, the latter rocks became protoliths for later metasomatic rocks. Most often, only one rock acts as protolith, and in this case, O1 = O2 = etc. In some particular cases, S and O may coincide. As a result of the indicated metasomatic event, rocks of several zones (A1, A2, etc.) were formed over the protolith. The bulk of component *x* was deposited into rock A1. As a consequence, its concentration in rock A1 has reached a value of $C_{x}^{A1}$. In turn, the bulk of component *y* was deposited into rock A2, reaching there the concentration of $C_{y}^{A2}$. The above set of processes (i.e., removing *x* and *y* components by the fluid and their redeposition in the rocks of A1 and A2 zones) is hereafter considered as “complementary metasomatic processes”. In the rocks of the other zones, the *x* and *y* components may also have been deposited, but significantly (by orders of magnitude) less abundant than in rocks A1 and A2. For the described case, isocon analysis can calculate the masses of rocks from zones A1 (*m*^A1^) and A2 (*m*^A2^) that can result from a given mass of the involved source rock (*m*^S^). The obtained estimate assumes that (a) the primary fluid did not contain or contained negligible *x* and *y* components, (b) the fluid carried out absolutely the entire volume of the *x* and *y* components from the source rock, and (c) this entire volume of *x* and *y* was deposited exclusively in rocks A1 and A2. At least the last two clauses are unattainable in natural objects. Therefore, the result of calculations for natural rocks represents the maximum possible values of *m*^A1^ and *m*^A2^. Provided that only the first condition is met, the true values of *m*^A1^ and *m*^A2^ must be lower. However, as shown below, the calculated values are sufficient for assessing the scale of mass transfer and interpreting the results for determining the conditions of geological processes.

The *m*^S^, *m*^A1^, and *m*^A2^ parameters are mathematically evaluated by performing several procedures. Isocon analysis calculates the absolute values of the *x* and *y* input into the rocks of zone A1. In terms of isocon analysis, these values have the notation $\Delta M_{x}^{A1}/M^{O1}\;$and $\Delta M_{y}^{A1}/M^{O1}$ [5]. For simplicity, we denote these parameters as *X*^A1^ and *Y*^A1^, respectively. Similarly, the absolute values of the input of these components into the rocks of the A2 zone are indicated as *X*^A2^ and *Y*^A2^. It is important to consider that the total value of the component input obtained using isocon analysis is determined relative to the mass of the original protolith rock and not the altered rock. In this regard, for a correct calculation, the absolute value of the component input must be normalized by the coefficient of mass change (*MC*), which is derived from the results of isocon analysis. The concentrations of the *x* and *y* components in the source rock are denoted as $C_{x}^{S}$ and $C_{y}^{S}$, respectively. For metasomatic processes that satisfy the above geological profile, the ratio of the parameters *m*^S^, *m*^A1^, and *m*^A2^ is given by the following equation system:

(4) $$m^{S}\;\times \;C_{x}^{S}\;=\;\frac{X^{A1}}{MC^{A1}}\;\times \;m^{A1}\;+\;\frac{X^{A2}}{MC^{A2}}\;\times \;m^{A2},$$

(5) $$m^{S}\;\times \;C_{y}^{S}\;=\;\frac{Y^{A1}}{MC^{A1}}\;\times \;m^{A1}+\frac{Y^{A2}}{MC^{A2}}\;\times \;m^{A2}.$$

Rearranging of equation (4) for a single value of the parameter *m*^S^ allows for expressing the value of *m*^A1^ as:

(6) $$m^{A1}\;=\;\frac{\left(C_{x}^{S}\;\times \;MC^{A2\;}-\;X^{A2}\;\times \;m^{A2}\right)\;\times \;MC^{A1}}{X^{A1}\;\times \;MC^{A2}}.$$

Substitution of Eq. (6) into Eq. (5) for a unit value of the parameter *m*^S^ yields:

(7) $$m^{S}\;\times \;C_{y}^{S}\;=\;\frac{Y^{A1}}{MC^{A1}}\;\times \;\frac{\left(C_{x}^{S}\;\times \;MC^{A2\;}-\;X^{A2}\;\times \;m^{A2}\right)\;\times \;MC^{A1}}{X^{A1\;}\times \;MC^{A2}}\;+\;\frac{Y^{A2}}{MC^{A2}}\;\times \;m^{A2}.$$

Transformation of equation (7) makes it possible to express *m*^A2^ via the known parameters $C_{x}^{S}$, $C_{y}^{S}$, *X*^A1^, *Y*^A1^, *X*^A2^, *Y*^A2^, and *MC*^A2^:

(8) $$m^{A2}\;=\;\frac{\left(C_{y}^{S}\times X^{A1}\;-\;C_{x}^{S}\;\times \;Y^{A1}\right)\;\times \;MC^{A2}}{\left(X^{A1}\;\times \;Y^{A2}-\;X^{A2}\;\times \;Y^{A1}\right)}.$$

In turn, substituting the solution of equation (8) into equation (6) allows for calculating the value of *m*^A1^. These simple calculations answer the question of the mass proportions of the rocks A1, A2, and S participating in the metasomatic process (in the ultimate case). Isocon analysis requires knowledge of rock densities; therefore, the available data a priori is sufficient to move from masses to volumes of rocks A1, A2, and S.

We considered a system with only two components (*x* and *y*) since this task was suitable for the object under investigation. However, if necessary and the geological grounds are available, the proposed approach can be extended to a more significant number of components and rocks of metasomatic zones, provided that the number of analyzed components and zones is equal.

### An example of applying the mass balance of complementary metasomatic processes and interpreting the results (method validation)

In the study of REE-rich carbonatites from the Petyayan-Vara area of the Vuoriyarvi complex (Kola region, NW Russia) [12], we found tracers of two processes that fit the above geological profile.

The first process (the relation with it is hereafter denoted by the symbol ') was manifested in the formation of baryte-rich (rock A1′) and ancylite-rich (rock A2′) carbonatites after burbankite-bearing magnesiocarbonatites (rock О1′). BaO and the sum of REE oxides (REE_2_O_3_) represent the *x′* and *y′* components, respectively. The source rock (S′) for these components was, again, burbankite-bearing magnesiocarbonatite (i.e., S′ = O1′). A similar genesis of rare earth carbonatites has been suggested for many other carbonatite complexes [13].

The second process (the relation to it is marked by the symbol ″) includes the formation of bastnäsite-rich (rock A1″) and strontianite-rich (rock A2″) carbonatites due to the accumulation of components released from the dissolved ancylite-rich carbonatites (rock-source S″). The *x″* component is REE_2_O_3_, and the *y″* component is SrO. A formation mechanism similar to that we assume for the mentioned Vuoriyarvi rocks has been described for the Bear Lodge complex, USA [14]. Notably, in Bear Lodge, the metasomatically altered rocks were also burbankite-bearing carbonatites. Based on the geological evidence for the Vuoriyarvi complex, we have chosen burbankite-bearing magnesiocarbonatites located near bastnäsite-rich (rock O1″) and strontianite-rich (rock O2″ = О1′) carbonatites as protoliths. Two protoliths were used for the experiment integrity because hundreds of meters of space apart bastnäsite-rich and strontianite-rich carbonatites. However, a numerical experiment showed that the use of one (common) protolith has almost no effect on the final result. We emphasize strong evidence that the primary fluids altering the source rocks contained very few *x* and *y* components under consideration [12,15,16]. We note that the considered components (Sr, Ba, and REEs) are abundant in carbonatites and very few in the host rocks. Thus, their introduction from an external source is unlikely, while their redistribution between the indicated types of carbonatites is confirmed by geological methods [12,15,16]. The proposed methodology is applicable to the specified geological profile of the processes. The chemical compositions and densities of all tested rocks are listed in Table 1. The results of mass balance using isocon analysis are shown in Table 2. In both tables, the parameters necessary for the calculation are marked. To carry out similar calculations for other tasks, the researcher will only need to (a) make sure that the analyzed processes meet the geological profile of the processes specified for this method; (b) select x and y components that were redistributed between rocks, but did not derive from an external source; (c) arrange the data as in Table 1; (d) perform an isocon analysis based on the data in Table 1 using the GeoIso [11] or its analogs; (e) present the obtained results as in Table 2; (f) determine parameters $C_{x}^{S}$, $C_{y}^{S}$, *X*^A1^, *Y*^A1^, *X*^A2^, *Y*^A2^, *MC*^A1^, and *MC*^A2^; (g) sequentially substitute these parameters into equations (8) and (6); and (h) compare the obtained *m*^S^, *m*^A1^, and *m*^A2^ values.

**Table 1:** Whole-rock compositions (wt.%) and density ρ (g/cm^3^) of carbonatites of the Petayan-Vara area (Vuoriyarvi massif) used for isocon analysis.

Sample:	15К05А-25.5a	15К10-00.0	15К05-24.5	15К05А-25.5c	15К10-02.5b	15К05А-15.0
Rock^1^:	BurC	BurC	BrtC	AncC	BasC	StrC
Role^2^:	S’=O1’=O2’=O2”	O1”	A1’	A2’=S”	A1”	A2”
SiO_2_	0.01	0.94	0.40	5.92	19.13	0.96
TiO_2_	0.06	0.10	0.07	0.04	0.10	0.12
Al_2_O_3_	0.04	0.11	0.02	0.69	0.07	0.22
Fe_2_O_3_	1.02	1.63	1.13	1.31	2.47	1.96
FeO	1.84	1.85	3.14	0.50	2.19	2.30
MnO	1.12	1.12	1.51	0.47	1.08	1.06
MgO	15.34	14.84	12.70	4.18	10.52	13.84
CaO	33.54	33.52	21.73	26.91	22.81	26.44
Na_2_O	0.14	0.10	0.05	0.09	0.10	0.08
K_2_O	0.04	0.05	0.27	0.01	0.01	0.01
Rb_2_O	0.00002	0.00005	0.00003	0.00004	0.00003	0.00005
P_2_O_5_	0.07	0.05	0.18	0.62	0.27	0.11
СО_2_	44.82	44.63	32.65	31.48	33.18	40.63
F	0.018	0.019	0.009	0.088	0.14	0.049
SO_3_	0.12	0.03	5.18	2.08	0.16	0.28
Cl	0.018	0.005	0.010	0.015	0.007	0.010
Н_2_О	0.20	0.24	0.28	0.26	0.3	0.04
SrO	0.95	0.50	0.79	7.40 ($C_{y}^{S^{\prime \prime }}$)	2.83	6.43
ВаО	0.39 ($C_{x}^{S^{\text{'}}})$^3^	0.07	17.06	4.85	0.36	0.82
ƩREE_2_O_3_	0.53 ($C_{y}^{S^{\prime }}$)	0.34	1.70	13.92 ($C_{x}^{S^{\prime \prime }}$)	4.99	2.24
Total	100.26	100.14	98.88	100.84	100.71	97.60
ρ^4^	2.93	2.86	3.03	3.06	2.87	2.87

1 Here and in Table 2, the following abbreviations designate carbonatites: BurC – burbankite-bearing magnesiocarbonatite; BrtC – baryte-rich carbonatite; AncC – ancylite-rich carbonatite; BasC – bastnäsite-rich carbonatite; StrC – strontianite-rich carbonatite.

2 Here and in Table 2, the roles of samples in model calculations are indicated by the following symbols: S – source rock; O1 – protolith for altered rocks of zone A1; O2 – protolith for altered rocks of zone A2; A1 – the altered rock of the zone A1; A2 – the altered rock of the zone A2. Explanations are given in the text.

3 Here and in Table 2, symbols in parentheses near the values used for calculations correspond to those in the text.

4 The rock density (ρ) was determined by hydrostatic weighing.

**Table 2:** Absolute values of gains and losses of components $\Delta M_{i}^{A}/M^{O}\;$(in g per 100 g of protolith) and the coefficients of mass change (parameter MC), calculated using isocon analysis in the GeoIso software [11] for models of carbonatite formation processes in the Petyayan-Vara field (Vuoriyarvi massif).

Component	First process:		Second process:	
	BurC (O1’) → BrtC (A1’)	BurC (O2’) → AncC (A2’)	BurC (O1”) → BasC (A1”)	BurC (O2”) → StrC (A2”)
SiO_2_	0.40	8.87	26.05	1.05
TiO_2_	0.01	0.00	0.04	0.07
Al_2_O_3_	–0.02	1.00	–0.01	0.20
Fe_2_O_3_	0.15	0.95	1.85	1.15
FeO	1.41	–1.09	1.24	0.71
MnO	0.44	–0.42	0.40	0.06
MgO	–2.20	–9.07	0.00	0.00
CaO	–11.05	6.83	–1.34	–4.23
Na_2_O	–0.09	–0.01	0.04	–0.05
K_2_O	0.24	–0.02	–0.03	–0.03
P_2_O_5_	0.12	0.86	0.33	0.05
СО_2_	–11.03	2.40	2.18	0.21
F	–0.01	0.11	0.18	0.04
SO_3_	5.24	3.00	0.20	0.19
Cl	–0.01	0.00	0.01	–0.01
SrO	–0.13	10.15	3.49 (*Y*^A1″^)	6.18 (*Y*^A2″^)
ВаО	17.27 (*X*^A1′^)	6.89 (*X*^A2′^)	0.43	0.53
ƩREE_2_O_3_	1.23 (*Y*^A1′^)	20.35 (*Y*^A2′^)	6.70 (*X*^A1″^)	1.95 (*X*^A2″^)
MC^1^	1.019 (*MC*^A1′^)	1.509 (*MC*^A2′^)	1.417 (*MC*^A1″^)	1.083 (*MC*^A2″^)
VC^2^	0%	44%	40%	13%
Immobile components^3^	V-const.	TiO_2_, Na_2_O, CaO, CO_2_	MgO, CO_2_	MgO, CO_2_

1 Mass change (MC) = $\left(\Sigma \Delta M_{i}^{A}/M^{O}+100\right)/100$.

2 The volume change (VC) values are from the results of isocon analysis performed using the GeoIso software.

3 Immobile components – the components selected as immobile when performing isocon analysis. Isocon analysis for the BurC (O1’) → BrtC (A1’) pair was carried out for a constant volume clause. For calculations, an auxiliary component V was added to the chemical components. Considering the difference in rock densities, at X(BurC) = 1.0, X(BrtC) = ρ(BurC)/ρ(BrtC) = 0.9662. The choice of the X as an immobile component in the GeoIso calculations allows performing isocon analysis for the condition of volume constancy (the calculated VC is 0%).

Table 2 additionally lists the components selected as immobile when performing calculations in the GeoIso program and assessing volume changes. These estimates are entirely consistent with our geological observations. Ancylite and bastnäsite carbonatites are breccias with a large volume of newly formed minerals cementing protolith fragments [12]. For these rocks, the estimate of volume change is high (≥+40%, see Table 2) and reflects the volume of cement. In strontianite carbonatites, newly formed mineral assemblages fill the veins [12]. Therefore, the volume change is less significant (+13%, see Table 2). Baryte carbonatites were formed due to pseudomorphic replacement and filling of cavities [12,16]. The isocon analysis was carried out for the volume constancy case for these rocks.

For the first process, the parameters $C_{x}^{S^{\prime }}$, $C_{y}^{S^{\prime }}$, *X*^A1′^, *Y*^A1′^, *X*^A2′^, *Y*^A2′^, and *MC*^A2′^ were substituted into equation (8), which yielded *m*^A2′^ = 0.038. Using this value and the $C_{x}^{S^{\prime }}$, *X*^A1′^, *X*^A2′^, *MC*^A1′^, and *MC*^A2′^ values in equation (6) gives a *m*^A1′^ = 0.013. Thus, the remobilization of 100 parts (by mass) of burbankite-bearing carbonatite can produce only 1.3 parts of baryte-rich carbonatite and 3.8 parts of ancylite carbonatite.

In the second process, the mass proportions of A1, A2, and S rocks were substantially different. Substitution of the corresponding parameters ($C_{x}^{S^{\prime \prime }},C_{y}^{S^{\prime \prime }}$, *X*^A1″^, *Y*^A1″^, *X*^A2″^, *Y*^A2″^, *MC*^A1″^, and *MC*^A2″^ from Tables 1 and 2) into equations (8) and (6) allowed estimating the values of *m*^A1″^ and *m*^A2″^ as 2.9 и 0.03 respectively. In sum, the remobilization of one part (by mass) of ancylite-rich carbonatites is sufficient for generating 2.9 parts of bastnäsite-rich carbonatites and 0.03 parts of strontianite-rich carbonatites.

Recall that we introduced some assumptions in the calculations, and the obtained values of *m*^A1^ and *m*^A2^ are thereby overestimated relative to *m*^S^. Due to the resulting uncertainty, verification of the results requires an additional deponent criterion to control how much this overestimation is critical for the model. This criterion can be the correlation between the calculated ratio of *m*^A1^: *m*^A2^ and the abundance of A1 and A2 rocks within the studied complex. The mass balance between rocks A1 and A2 in both considered cases is consistent with our field observations. For the first process, *m*^A1′^: *m*^A2′^ ≈ 3:1; and for the second process, *m*^A1″^: *m*^A2″^ ≈ 100:1. Field observations in the Petyayan-Vara field show that baryte-rich carbonatites are several times more abundant than ancylite-rich carbonatites. Also, bastnäsite-rich carbonatites compose significant volumes of carbonatite veins, whereas strontianite-rich carbonatites occur extremely locally.

We believe that any numerical results of mass balance studies and related methods (geochemical, isotopic, etc.) should be treated with some caution since they are greatly influenced by the choice of samples used for the model (variability, closeness to the average composition, etc.). However, qualitative data is often sufficient for geological interpretations and understanding the direction of the processes. In our methodology, valuable geological information is provided by the qualitative relations between the *m*^S^, *m*^A1^, and *m*^A2^ parameters. For the first of the considered processes, *m*^S^′ ≫ (*m*^A1^′ + *m*^A2^′) (*m*^S^′: *m^A1^*′: *m*^A2^′ = 1: 0.013: 0.038), for the second process, *m*^S^″ ≈ (*m*^A1^″ + *m*^A2^″) (*m*^S^″: *m^A1^*″: *m*^A2^″ = 1: 2.9: 0.03). The former inequality suggests that the process could only proceed on a large scale, involving a large volume of source rock. The latter inequality indicates a process that could occur locally. In the second case, the mass of bastnäsite-rich carbonatites was several times greater than the mass of remobilized rock (ancylite-rich carbonatites), which indicates the involvement of an additional (external) source. The main contribution to the mass change during the formation of bastnäsite carbonatites is made by the non-carbonatite SiO_2_ component (see Table 2), which indicates a significant input from the host aluminosilicate rocks. These interpretations fully agree with the available geological data [16].

## Conclusion

This paper demonstrates the use of isocon analysis for estimating the mass proportions between the source rock and complementary metasomatic rocks generated from the material remobilized from this source rock. This method requires a robust geological justification like any mass balance investigations of metasomatism and other mathematical methods in geology. One of the geological tools for checking the correctness of isocon analysis results is comparing the calculated mass proportions of metasomatic rocks with their prevalence within the studied object. The algorithm is quite simple:

(a) determine whether the analyzed processes meet the geological profile to which this method is applicable (the geological profile as detailed above);
(b) select *x* and *y* components that were redistributed by a fluid between rocks (not from an external source);
(c) summarize data on the chemical composition of rocks and their densities in the manner of Table 1;
(d) perform isocon analysis based on these data using the GeoIso software [11] or its analogs, considering the geological prerequisites for immobile components or compliance with the volume constancy condition;
(e) bring the results into a table similar to Table 2;
(f) determine (based on the selected x and y components) the $C_{x}^{S}$, $C_{y}^{S}$, *X*^A1^, *Y*^A1^, *X*^A2^, *Y*^A2^, *MC*^A1^, and *MC*^A2^, parameters, necessary for further calculations;
(g) substitute these parameters into equations (8) and (6);
(h) compare the obtained values of *m*^S^, *m*^A1^, and *m*^A2^ and carry out a geological interpretation of the results.

## Declaration of Competing Interest

The authors declare that they have no known competing financial interests or personal relationships that could have appeared to influence the work reported in this paper.
